# Dietary fatty acids differentially affect secretion of pro-inflammatory cytokines in human THP-1 monocytes

**DOI:** 10.1038/s41598-023-32710-5

**Published:** 2023-04-04

**Authors:** Hao-Chang Hung, Sheng-Feng Tsai, Hsuan-Wen Chou, Ming-Jun Tsai, Pei-Ling Hsu, Yu-Min Kuo

**Affiliations:** 1 Division of Endocrinology and Metabolism, Department of Internal Medicine, Kaohsiung Veterans General Hospital, Kaohsiung, 81362 Taiwan; 2 Department of Cell Biology and Anatomy, College of Medicine, National Cheng Kung University, 1 Ta Hsueh Road, Tainan, 70101 Taiwan; 3 Institute of Basic Medical Sciences, College of Medicine, National Cheng Kung University, Tainan, 70101 Taiwan; 4 Division of Endocrinology and Metabolism, Department of Internal Medicine, National Cheng Kung University Hospital, College of Medicine, National Cheng Kung University, Tainan, 70403 Taiwan; 5 School of Medicine, College of Medicine, China Medical University, Taichung, 40402 Taiwan; 6 Department of Neurology, China Medical University Hospital, Taichung, 40447 Taiwan; 7 Department of Neurology, An-Nan-Hospital, China Medical University, Tainan, 709204 Taiwan; 8 Department of Anatomy, School of Medicine, College of Medicine, Kaohsiung Medical University, 100 Shih-Chuan 1St Road, Kaohsiung, 80708 Taiwan; 9 Department of Medical Research, Kaohsiung Medical University Hospital, Kaohsiung, 80708 Taiwan

**Keywords:** Cytokines, Lipids

## Abstract

Monocytes are a major population of circulating immune cells that play a crucial role in producing pro-inflammatory cytokines in the body. The actions of monocytes are known to be influenced by the combinations and concentrations of certain fatty acids (FAs) in blood and dietary fats. However, systemic comparisons of the effects of FAs on cytokine secretion by monocytes have not be performed. In this study, we compared how six saturated FAs (SFAs), two monounsaturated FAs (MUFAs), and seven polyunsaturated FAs (PUFAs) modulate human THP-1 monocyte secretion of TNF, IL-1β, and IL-6 in the absence or presence of lipopolysaccharide. SFAs generally stimulated resting THP-1 cells to secrete pro-inflammatory cytokines, with stearic acid being the most potent species. In contrast, MUFAs and PUFAs inhibited lipopolysaccharide-induced secretion of pro-inflammatory cytokines. Interestingly, the inhibitory potentials of MUFAs and PUFAs followed U-shaped (TNF and IL-1β) or inverted U-shaped (IL-6) dose–response curves. Among the MUFAs and PUFAs that were analyzed, docosahexaenoic acid (C22:6 n-3) exhibited the largest number of double bonds and was found to be the most potent anti-inflammatory compound. Together, our findings reveal that the chemical compositions and concentrations of dietary FAs are key factors in the intricate regulation of monocyte-mediated inflammation.

## Introduction

Inflammation is an essential protective response that helps an organism to resolve infections and injuries^1,2^. However, in certain circumstances, the acute inflammatory response may progress to a persistent non-resolving response that becomes harmful to the host^3^. Chronic systemic inflammation can lead to a breakdown in immune tolerance^4–6^ and increase the risks of various non-communicable diseases, including cancer, cardiovascular disease, metabolic disorders and neurodegenerative diseases^7–12^. Prolonged inflammation can also weaken the immune system, leading to increased risk of infections and decreased response to vaccination^13–15^. Additionally, early-life chronic inflammation can have serious developmental consequences that raise an individual’s lifetime risk of developing non-communicable diseases^16–19^. Notably, chronic inflammation-related diseases now contribute to over 50% of all deaths^20,21^. Nevertheless, inflammation is necessary for survival, and maintaining a balance of cellular and molecular inflammation mediators is crucial for many essential homeostatic processes, including tissue remodeling, metabolism, and nervous system function^22^. Thus, there is an urgent need for new clinical strategies to finely control the inflammation state.

The compositions of dietary fats greatly affect the profiles and concentrations of fatty acids (FAs) in the blood^23–27^, and these circulating FAs are known to modulate inflammatory status in humans and animals^28^. FAs are generally categorized into three classes according to their chemical structures (i.e., number of double bonds): saturated FAs (SFAs), monounsaturated FAs (MUFAs), and polyunsaturated FAS (PUFAs). In general, SFAs are regarded as pro-inflammatory factors^29–31^, whereas MUFAs and PUFAs appear to function as anti-inflammatory mediators^32–35^. As such, it is widely assumed that dietary intake of different classes of fats will alter the circulating FA profiles and differentially affect the activation status of circulating immune cells. Yet, systematic explorations of how different dietary fatty acids affect immune cells are still lacking.

Monocytes are a major population of circulating immune cells that can be recruited from the bloodstream to peripheral tissues, where they differentiate into either macrophages or dendritic cells to support both innate and adaptive immune responses^36^. Upon activation, macrophages in different tissues respond to certain microenvironmental stimuli by taking on M1 or M2 polarizations^37–39^. In infected or injured tissues, macrophages first polarize to an M1 state in order to eliminate pathogens; then, the cells take on an M2 polarization and function to repair tissue damage^39^. Three main categories of stimuli have been identified as inducers of M1 macrophages, including interferon-γ, pathogens, and granulocyte macrophage colony-stimulating factor^37^. Meanwhile, M2 macrophages can be induced by interleukin (IL)-4, IL-10, glucocorticoids, and macrophage colony-stimulating factor^37^. The polarization of macrophages is most often characterized by detection of cell surface or secreted markers. For instance, M1 macrophages express high levels of cluster of differentiation (CD)16/32, CD80, and CD86 and are capable of secreting pro-inflammatory cytokines such as tumor necrosis factor (TNF), IL-1β, IL-6, IL-12, and IL-18^37,39,40^. In contrast, M2 macrophages express high levels of arginase-1, CD206, and anti-inflammatory cytokines and chemokines such as IL-10 and CCL17 and CCL22^37,39^. Pro-inflammatory cytokines produced by monocytes and M1 macrophages (e.g., TNF, IL-1β and IL-6) may contribute to the development of non-resolving inflammation and play important roles in the pathophysiology of various non-communicable inflammation-related diseases^41^. Importantly, the production of TNF, IL-1β, and IL-6 by monocytes is known to be substantially influenced by certain FAs^42–44^.

The goal of this study was to investigate how different classes of dietary FAs might influence the production of TNF, IL-1β, and IL-6 in THP-1 cells, a widely used human monocyte cell line. The concentrations of FAs used in our experiments (0–500 µM) reflected typical levels of circulating FAs in humans^45,46^. Initially, we compared the impact of 15 different FAs (comprising of six SFAs, two MUFAs, and seven PUFAs as listed in Table 1) on the secretion of targeted cytokines by THP-1 cells. We selected the 15 FAs for our experiments based on their richness in diet, capacity to be detected in blood, and known roles in the pathogenesis of metabolic disorders^47–50^. In further experiments, THP-1 cells were treated with FAs in the presence of lipopolysaccharide (LPS) to examine the potential anti-inflammatory functions of the FAs.

**Table 1:** List of selected dietary fatty acids used in this study.

Common name	IUPAC name	Lipid numbers
caprylic acid	octanoic acid	C8:0
capric acid	decanoic acid	C10:0
undecylic acid	undecanoic acid	C11:0
lauric acid	dodecanoic acid	C12:0
palmitic acid	hexadecanoic acid	C16:0
stearic acid	octadecanoic acid	C18:0
palmitoleic acid	(9Z)-hexadec-9-enoic acid	C16:1 (n-7)
oleic acid	(9Z)-octadec-9-enoic acid	C18:1 (n-9)
linoleic acid	(9Z,12Z)-octadeca-9,12-dienoic acid	C18:2 (n-6)
α-linolenic acid	(9Z,12Z,15Z)-octadeca-9,12,15-trienoic acid	C18:3 (n-3)
γ-linolenic acid	(6Z,9Z,12Z)-octadeca-6,9,12-trienoic acid	C18:3 (n-6)
arachidonic acid	(6Z,9Z,12Z)-octadeca-6,9,12-trienoic acid	C20:4 (n-6)
eicosapentaenoic acid (EPA)	(5Z,8Z,11Z,14Z,17Z)-eicosa-5,8,11,14,17-pentenoic acid	C20:5 (n-3)
docosapentaenoic acid (DPA)	(7Z,10Z,13Z,16Z,19Z)-docosapentaenoic acid	C22:5 (n-3)
docosahexaenoic acid (DHA)	(4Z,7Z,10Z,13Z,16Z,19Z)-docosa-4,7,10,13,16,19-hexaenoic acid	C22:6 (n-3)

## Results

### Effects of FAs on secretion of TNF by human THP-1 monocytes

**Figure 1:**
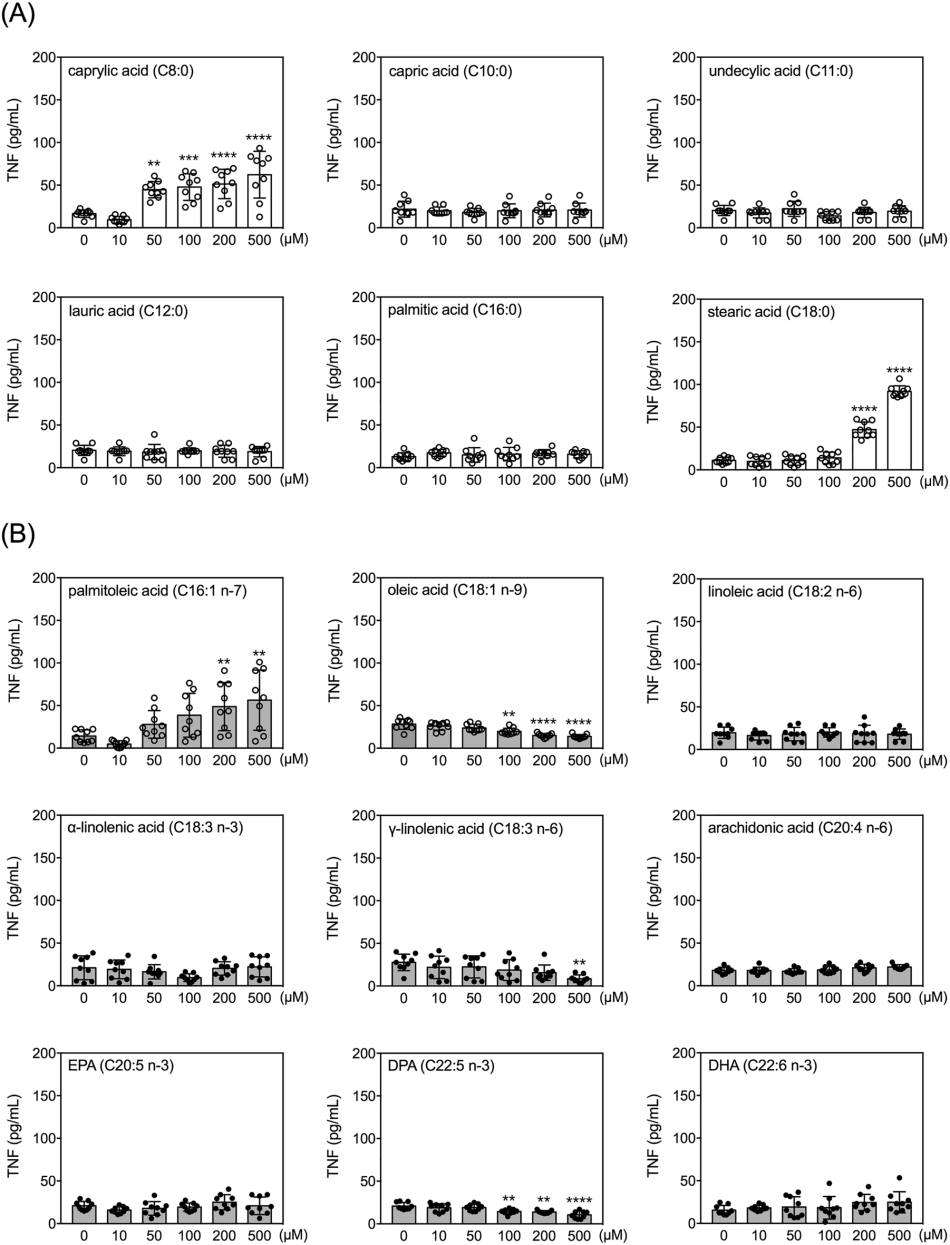
Effects of fatty acids on TNF secretion by human THP-1 monocytes. Quantitative results of levels of TNF in the conditioned media of THP-1 cells treated with selected FAs at different doses for 24 h. Data are presented as mean ± standard deviation. **p < 0.01, ***p < 0.001, ****p < 0.0001, versus 0 µM vehicle group, Dunnett’s multiple comparisons after one-way ANOVAs. n = 9.

The viability of THP-1 cells was not significantly affected by incubation with any of the 15 FAs up to 24 h at any of the tested concentrations (up to 500 µM) (Supplementary Table 1). However, six of the 15 FAs significantly and dose-dependently changed the levels of TNF in the conditioned media of THP-1 cells (Fig. 1). Among these six FAs, caprylic acid, stearic acid and palmitoleic acid increased TNF, while oleic acid, γ-linolenic acid, and DPA decreased levels of secreted TNF (Fig. 1). Among the three FAs that increased TNF levels, caprylic acid had the lowest effective dose (50 µM). Oleic acid and DPA treatments decreased TNF levels at concentrations of 100 µM and higher (Fig. 1).

**Figure 2:**
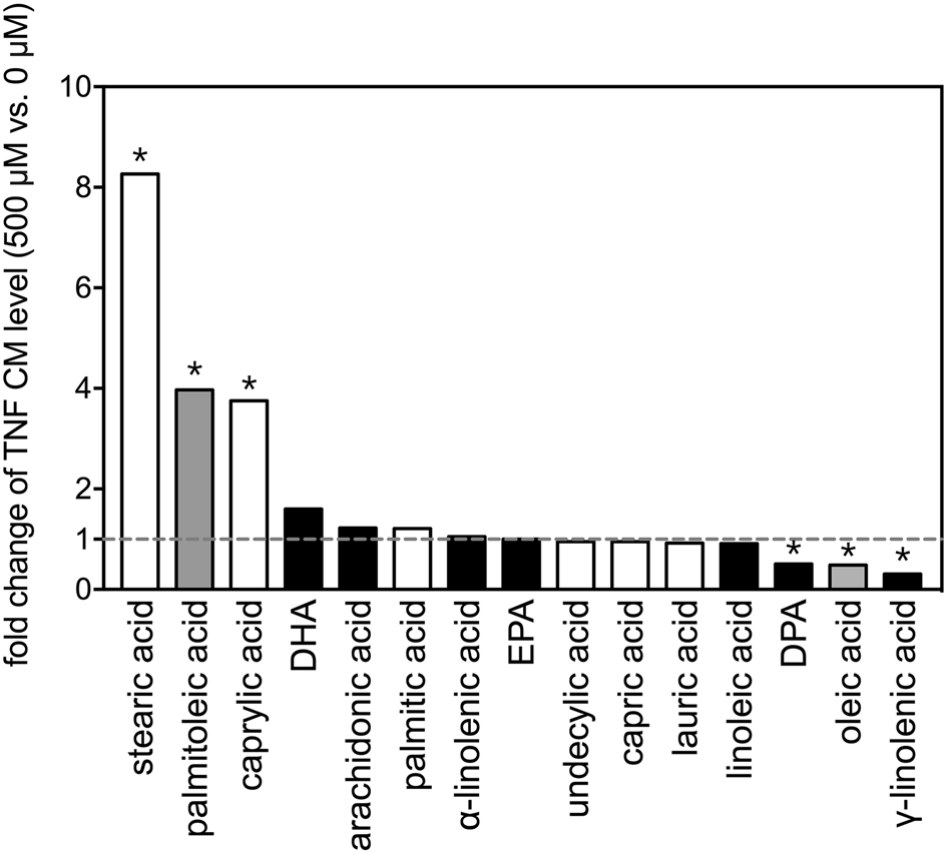
Fold-changes of TNF levels after treatment with selected fatty acids. Fold changes in levels of TNF in the conditioned media between 500 µM and 0 µM (vehicle control) of the 15 FAs after incubation with THP-1 cells for 24 h. The asterisks indicate significance in *post-hoc* comparison (500 µM vs. 0 µM) shown in Fig. 1.

Fold-changes in TNF levels were calculated from cultures treated with 500 µM and 0 µM (vehicle control) of each of the 15 FAs (Fig. 2). Stearic acid induced the largest fold-change, followed by palmitoleic acid and caprylic acid. Among the three FAs that decreased TNF levels, γ-linolenic acid most potently decreased the level of TNF (to about 30% of the vehicle control group), followed by oleic acid and DPA (Fig. 2).

### Effects of FAs on secretion of IL-1β by human THP-1 monocytes

**Figure 3:**
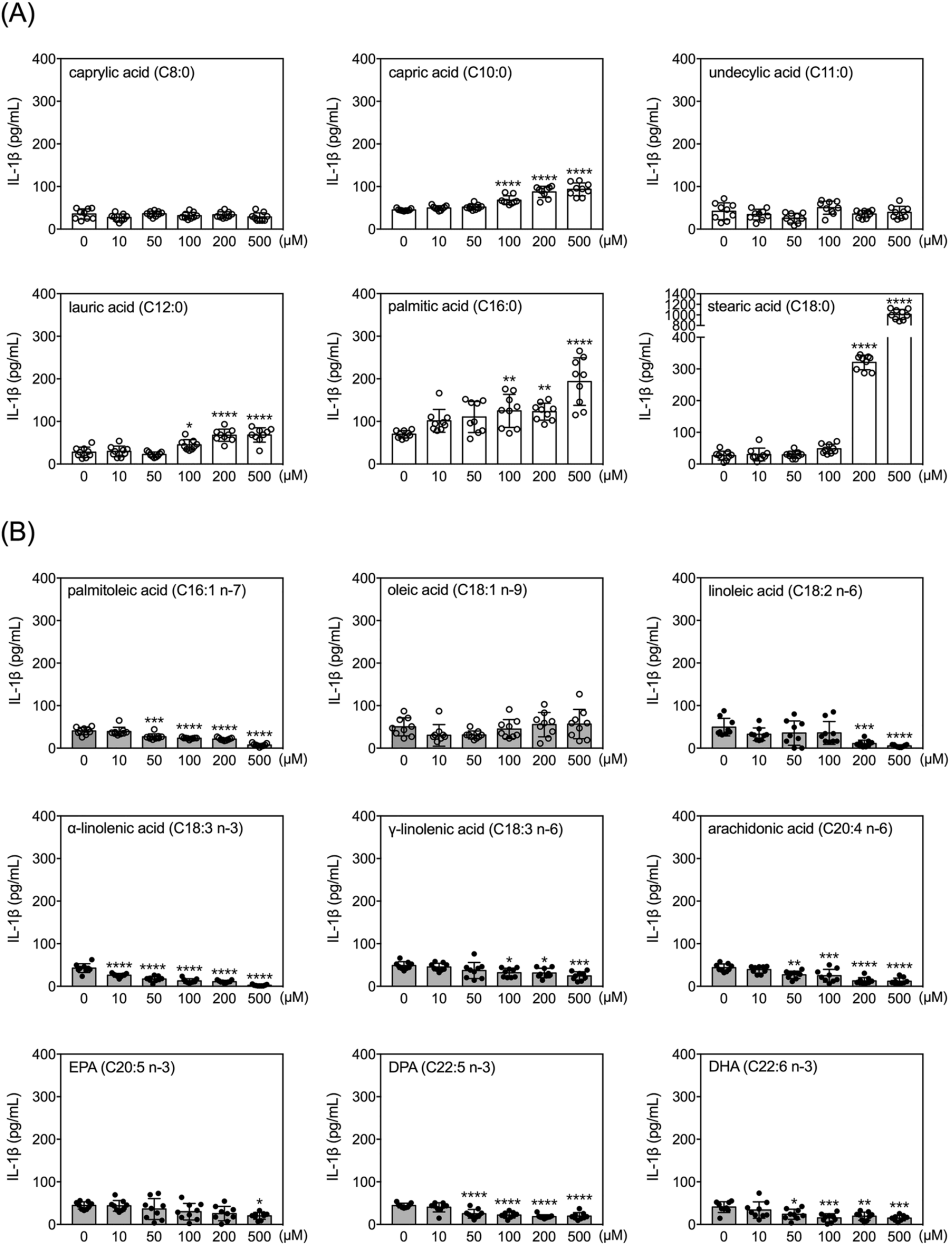
Effects of fatty acids on IL-1β secretion by human THP-1 monocytes. Quantitative results of levels of IL-1β in the conditioned media of THP-1 cells treated with selected FAs at different doses for 24 h. Data are presented as mean ± standard deviation. *p < 0.05, **p < 0.01, ***p < 0.001, ****p < 0.0001, versus 0 µM vehicle group, Dunnett’s multiple comparisons after one-way ANOVAs. n = 9.

Within the concentration range tested, capric acid, lauric acid, palmitic acid, and stearic acid increased IL-1β in conditioned media. Palmitoleic acid, linoleic acid, α-linolenic acid, γ-linolenic acid, arachidonic acid, EPA, DPA, and DHA decreased the levels of IL-1β in the conditioned media (Fig. 3). All of these effects were dose dependent. Three FAs (caprylic acid, undecylic acid, and oleic acid) did not significantly affect IL-1β levels in THP-1 conditioned media. Among the four FAs that increased IL-1β levels, caprylic acid, lauric acid, and palmitic acid were effective at concentrations of 100 µM or higher. Among the eight FAs that decreased IL-1β levels, α-linolenic acid had the lowest effective dose (10 µM), followed by palmitoleic acid, arachidonic acid, DPA and DHA (effective dose, 50 µM) (Fig. 3).

**Figure 4:**
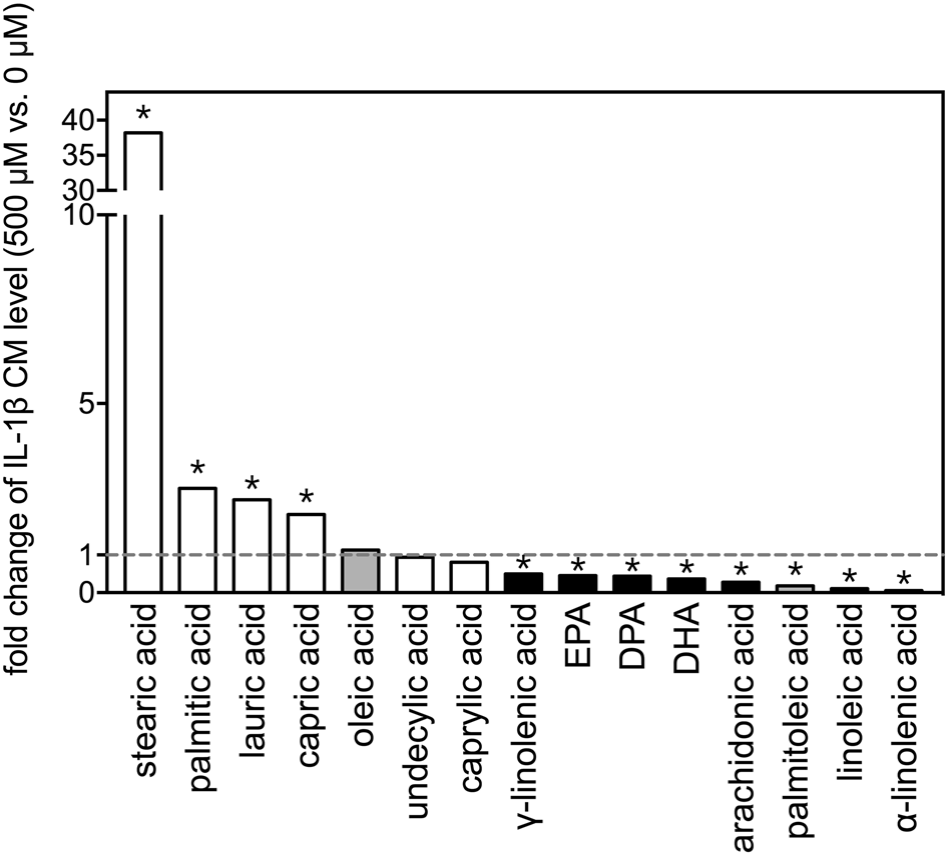
Fold-changes of IL-1β levels after treatment with selected fatty acids. Fold changes in levels of IL-1β in the conditioned media between 500 µM and 0 µM (vehicle control) of the 15 FAs after incubation with THP-1 cells for 24 h. The asterisks indicate significance in *post-hoc* comparison (500 µM vs. 0 µM) shown in Fig. 3.

The fold-changes in IL-1β levels (comparing 500 µM to 0 µM) for the 15 FAs are shown in Fig. 4. Stearic acid dramatically increased levels of IL-1β in the conditioned medium to more than 30-fold the level seen in the vehicle control group. Meanwhile, α-linolenic acid and linoleic acid potently decreased levels of IL-1β to about 10% of that in the vehicle control group.

### Effects of FAs on secretion of IL-6 by human THP-1 monocytes

**Figure 5:**
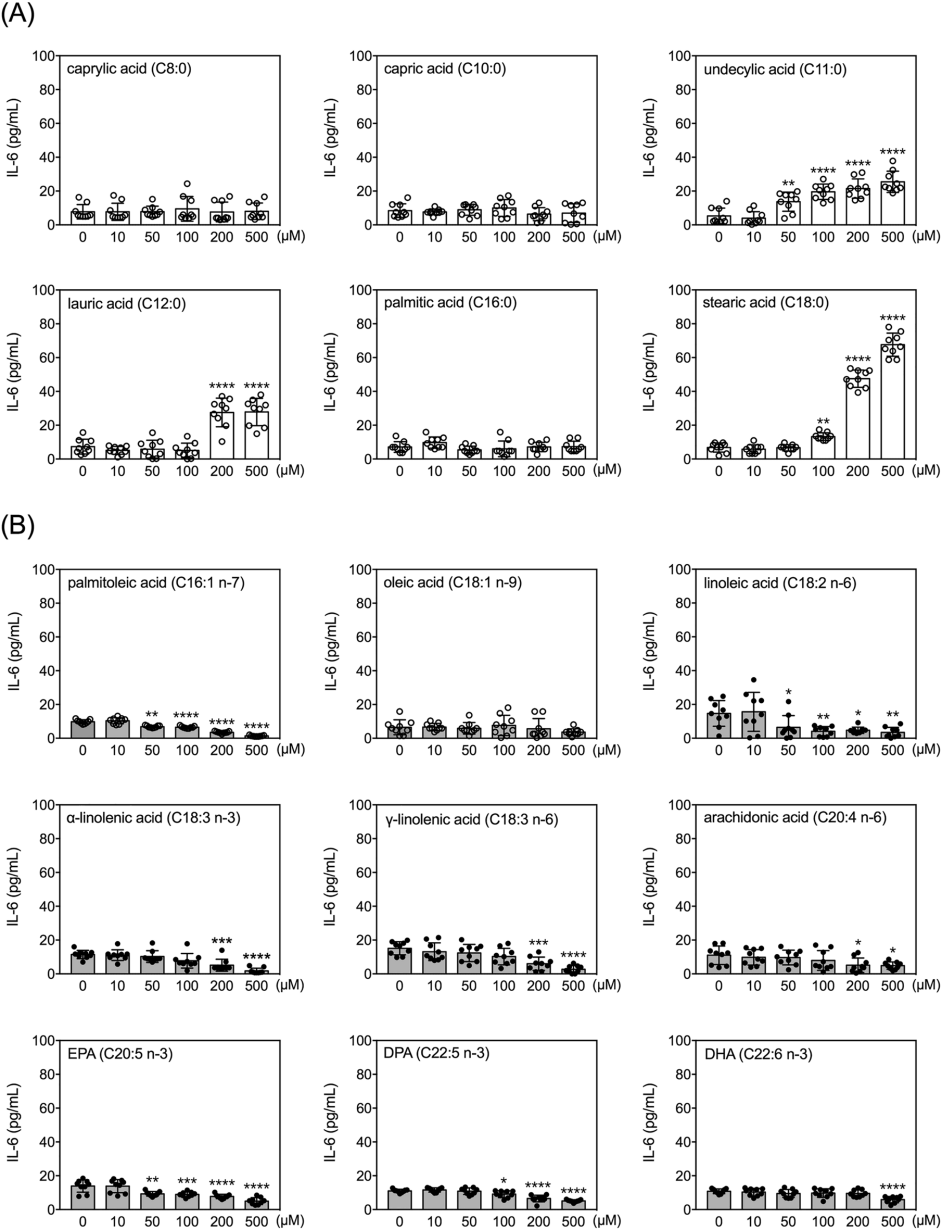
Effects of fatty acids on IL-6 secretion by human THP-1 monocytes. Quantitative results of levels of IL-6 in the conditioned media of THP-1 cells treated with selected FAs at different doses for 24 h. Data are presented as mean ± standard deviation. *p < 0.05, **p < 0.01, ***p < 0.001, ****p < 0.0001, versus 0 µM vehicle group, Dunnett’s multiple comparisons after one-way ANOVAs. n = 9.

Within the tested concentration range, undecylic acid, lauric acid, and stearic acid dose-dependently increased levels of IL-6 in conditioned media, while palmitoleic acid, linoleic acid, α-linolenic acid, γ-linolenic acid, arachidonic acid, EPA, DPA and DHA all decreased the levels of IL-6 (Fig. 5). Among the three FAs that increased IL-6 levels, undecylic acid had the lowest effective dose (50 µM). Regarding the FAs that decreased IL-6 levels, palmitoleic acid, linoleic acid and EPA were effective at 50 µM and higher concentrations (Fig. 5).

**Figure 6:**
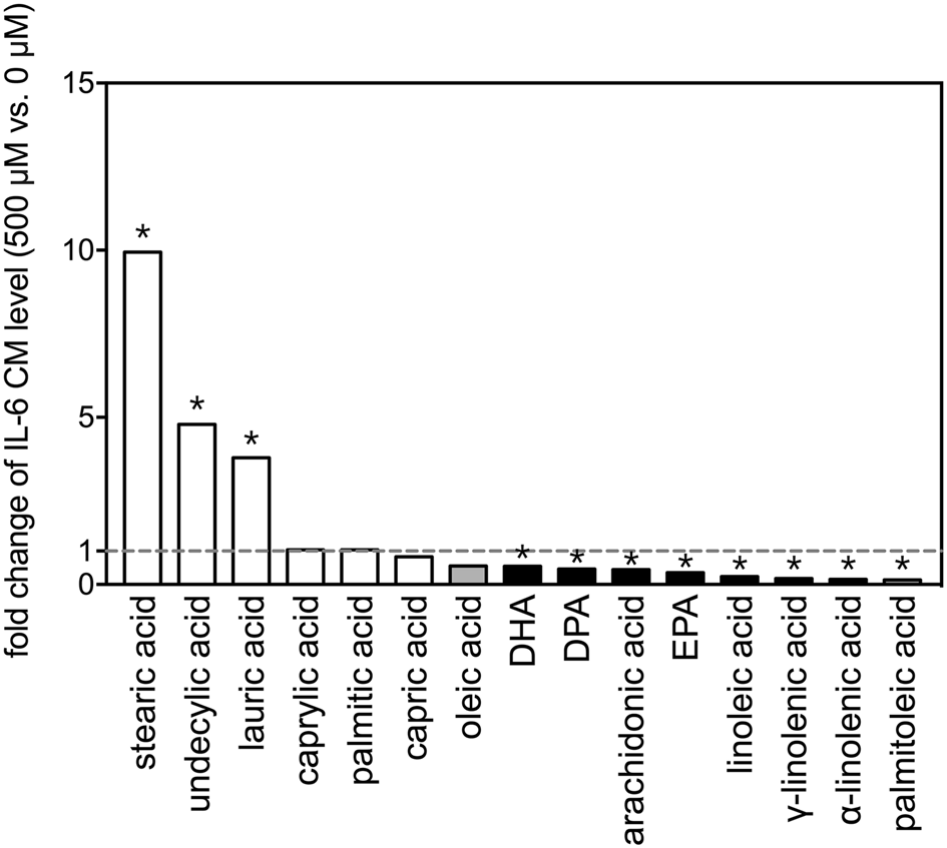
Fold-changes of IL-6 levels after treatment with selected fatty acids. Fold changes in levels of IL-6 in the conditioned media between 500 µM and 0 µM (vehicle control) of the 15 FAs after incubation with THP-1 cells for 24 h. The asterisks indicate significance in *post-hoc* comparison (500 µM vs. 0 µM) shown in Fig. 5.

Comparing the 500 µM dose to respective vehicle controls (0 µM), stearic acid induced the largest increase in IL-6 level (~ tenfold) (Fig. 6), whereas palmitoleic acid, α-linolenic acid, and γ-linolenic acid all reduced the levels of IL-6 to less than 20% of those in the respective vehicle control groups (Fig. 6).

### Effects of FAs on secretion of TNF by LPS-treated human THP-1 monocytes

**Figure 7:**
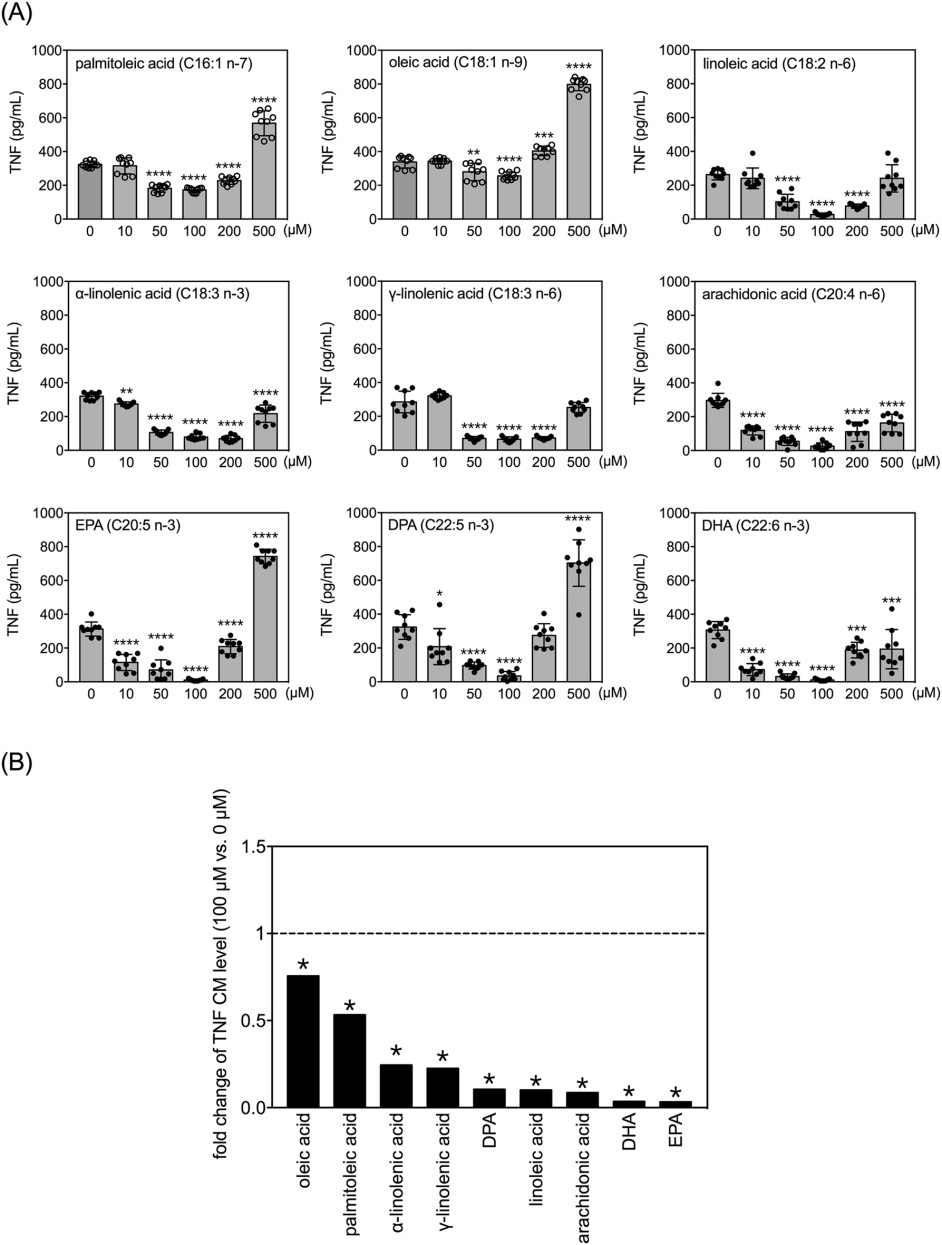
Effects of selected MUFAs and PUFAs on TNF secretion by LPS-treated human THP-1 monocytes. **(A)** Quantitative results of levels of TNF in the conditioned media of THP-1 cells treated with selected FAs at different doses and 100 ng/mL of LPS for 24 h. Data are presented as mean ± standard deviation. *p < 0.05, **p < 0.01, ***p < 0.001, ****p < 0.0001 verses 0 µM vehicle group, Dunnett’s multiple comparisons after one-way ANOVAs. n = 9. **(B)** Fold changes in levels of TNF in the conditioned media between 100 µM and 0 µM (vehicle control) among the selected fatty acids with LPS for 24 h. The asterisks indicate significance in *post-hoc* comparison (100 µM vs. 0 µM) shown in panel (**A**).

Since two MUFAs and seven PUFAs were capable of diminishing the levels of TNF, IL-1β and IL-6 in conditioned media of resting THP-1 monocytes (Figs. 1, 3, and 5), we next wanted to examine how FAs affect the three pro-inflammatory cytokines after LPS treatment. THP-1 cells were treated with LPS at 100 ng/mL, which significantly increased the level of TNF in the conditioned medium (Fig. 7A compared to Fig. 1). Interestingly, both MUFAs and all seven PUFAs affected LPS-induced TNF production with a U-shaped dose–response curve (Fig. 7A). All nine FAs strongly inhibited LPS-induced TNF production when treated at 100 µM (Fig. 7A). However, these inhibitory effects became either less pronounced (i.e., α-linolenic acid, arachidonic acid, and DHA), insignificant (i.e., linoleic acid and γ-linolenic acid), or even reversed (i.e., palmitoleic acid, oleic acid, EPA, and DPA) at concentrations of 500 µM (Fig. 7A).

Fold-changes in TNF levels between the most effective dose (100 µM) and vehicle control were compared across the nine FAs (Fig. 7B). EPA and DHA elicited the strongest inhibition of LPS-induced TNF production (< 5% of the vehicle control group), followed by arachidonic acid, linoleic acid and DPA (~ 10% of the vehicle control group).

### Effects of FAs on secretion of IL-1β by LPS-treated human THP-1 monocytes

**Figure 8:**
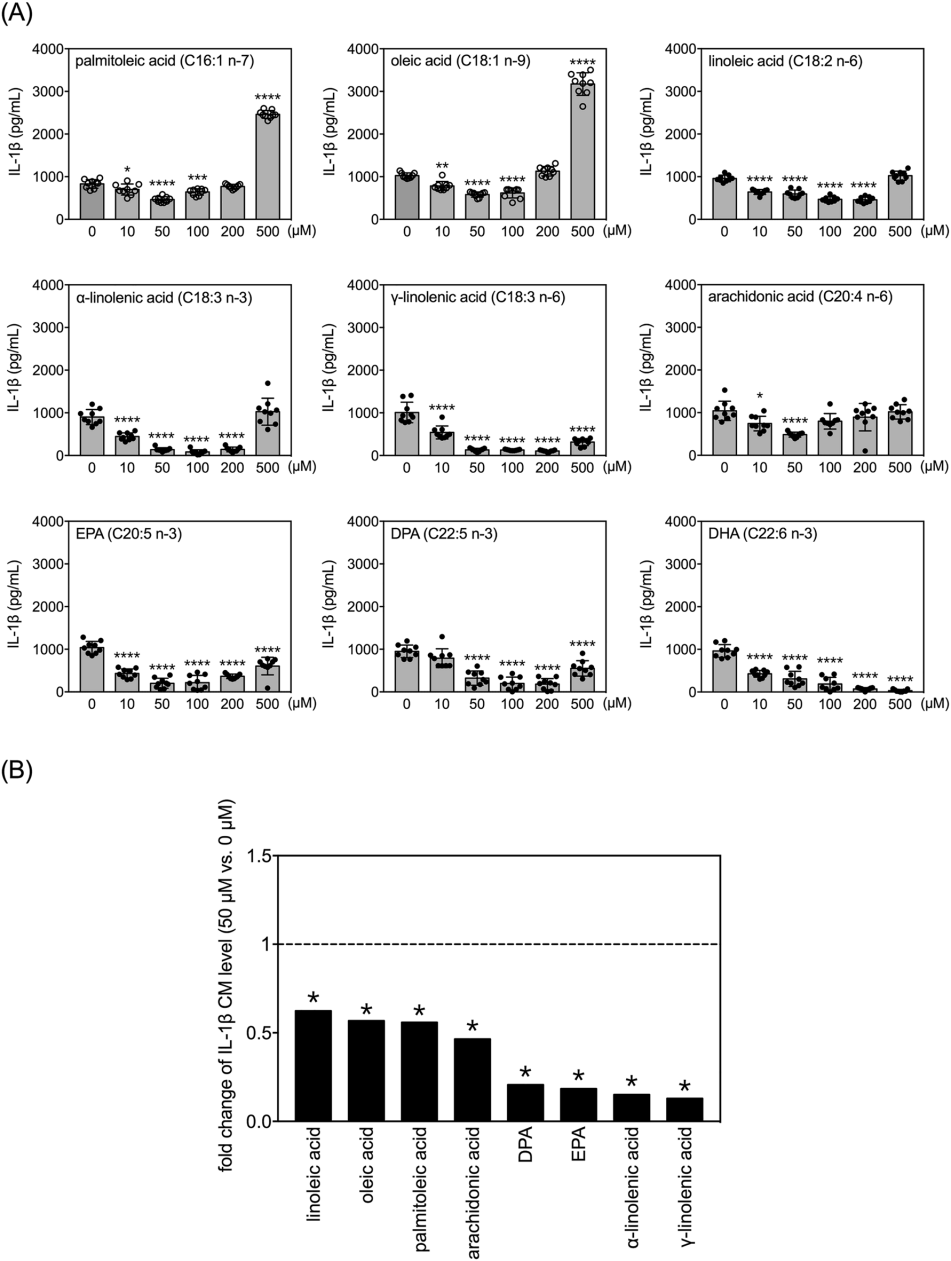
Effects of selected MUFAs and PUFAs on IL-1β secretion by LPS-treated human THP-1 monocytes. **(A)** Quantitative results of levels of IL-1β in the conditioned media of THP-1 cells treated with selected FAs at different doses and 100 ng/mL of LPS for 24 h. Data are presented as mean ± standard deviation. *p < 0.05, **p < 0.01, ***p < 0.001, ****p < 0.0001 verses 0 µM vehicle group, Dunnett’s multiple comparisons after one-way ANOVAs. n = 9. **(B)** Fold changes in levels of IL-1β in the conditioned media between 50 µM and 0 µM (vehicle control) among the selected fatty acids with LPS for 24 h. The asterisks indicate significance in *post-hoc* comparison (50 µM vs. 0 µM) shown in panel (**A**).

Treatment with 100 ng/mL LPS also significantly increased levels of IL-1β in the conditioned medium of THP-1 monocytes (Fig. 8A vs. Figure 3). Similar to the results of TNF measurements, all of the examined MUFAs and PUFAs except DHA regulated LPS-induced IL-1β production with U-shaped dose–response curves (Fig. 8A). Within the tested concentration range, DHA dose-dependently repressed LPS-induced elevation of IL-1β to less than 5% of the vehicle control group (Fig. 8A). The other eight FAs inhibited LPS-induced IL-1β production most potently at either 50 or 100 µM (Fig. 8A).

For comparison of fold-changes in IL-1β levels, we selected the 50 µM dose for normalization to the respective vehicle control groups (Fig. 8B). Among the two MUFAs and six PUFAs (excluding DHA) that exhibited effects, γ-linolenic acid, α-linolenic acid, EPA and DPA most strongly inhibited LPS-induced IL-1β secretion to approximately 20% or less of the level seen in the respective vehicle control group.

### Effects of FAs on secretion of IL-6 by LPS-treated human THP-1 monocytes

**Figure 9:**
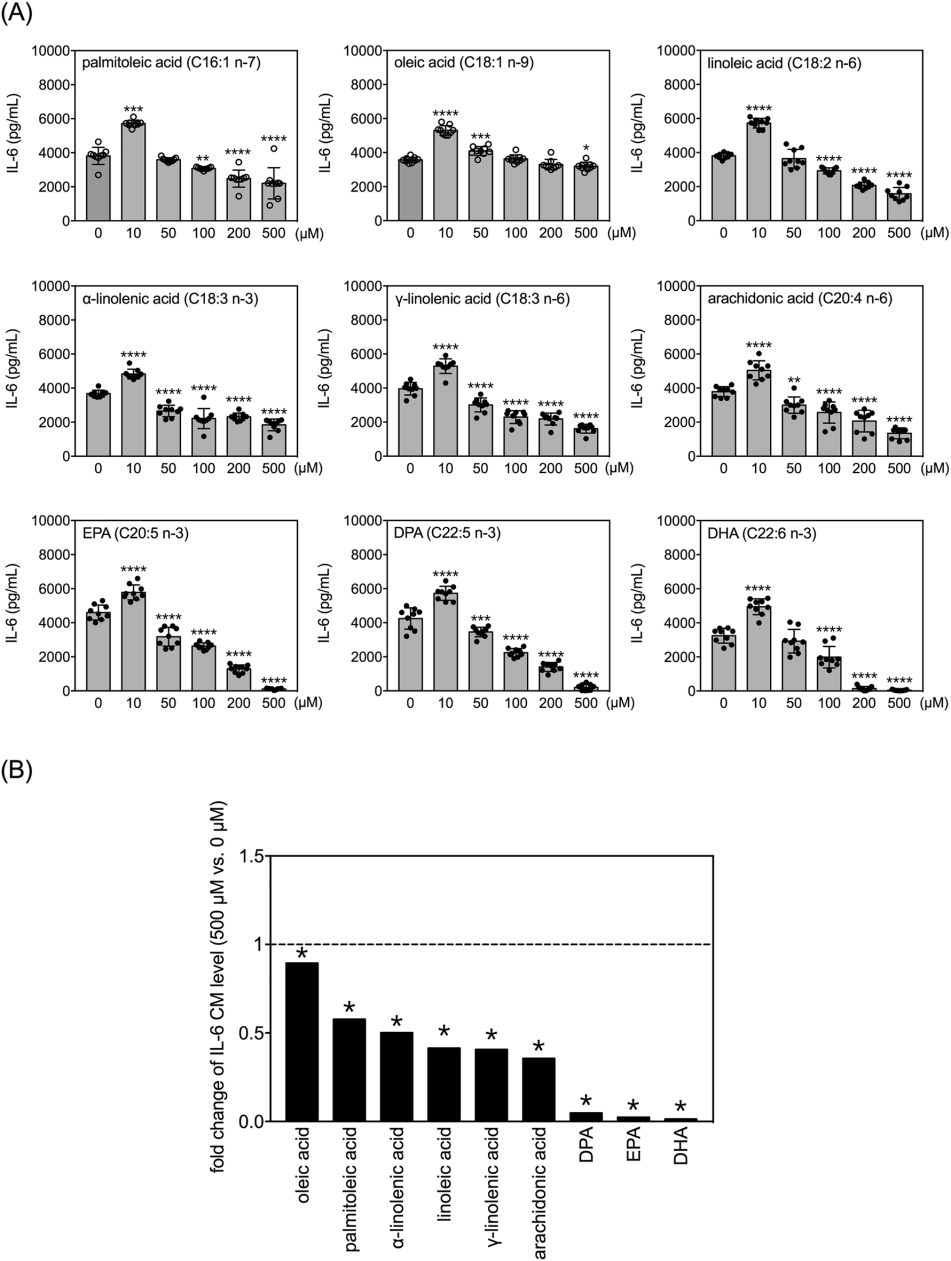
Effects of selected MUFAs and PUFAs on IL-6 secretion by LPS-treated human THP-1 monocytes. **(A)** Quantitative results of levels of IL-6 in the conditioned media of THP-1 cells treated with selected FAs at different doses and 100 ng/mL of LPS for 24 h. Data are presented as mean ± standard deviation. *p < 0.05, **p < 0.01, ***p < 0.001, ****p < 0.0001 verses 0 µM vehicle group, Dunnett’s multiple comparisons after one-way ANOVAs. n = 9. **(B)** Fold changes in levels of IL-6 in the conditioned media between 50 µM and 0 µM (vehicle control) among the selected fatty acids with LPS for 24 h. The asterisks indicate significance in *post-hoc* comparison (50 µM vs. 0 µM) shown in panel (**A**).

LPS treatment (100 ng/mL) strongly increased levels of IL-6 in the conditioned media of THP-1 monocytes (Fig. 9A vs. Fig. 5). Unlike the U-shaped dose–response curves observed for TNF and IL-1β, the nine FAs generally regulated LPS-induced IL-6 secretion according to inverted U-shaped dose–response curves (Fig. 9A). All nine FAs potentiated LPS-induced IL-6 production at 10 µM (Fig. 9A). However, at concentrations higher than 100 µM, eight of the nine FAs (except oleic acid) dose-dependently inhibited LPS-induced IL-6 production (Fig. 9A).

We selected the most potent effective dose within the selected concentration range (500 µM) to compare fold changes in IL-6 levels to respective vehicle controls (Fig. 9B). DHA, EPA and DPA strongly inhibited the LPS-induced IL-6 levels to less than 5% of their respective vehicle control groups.

## Discussion

Several studies have explored the differences between various FAs in terms of their impacts on innate immune cell responses. However, to the best of our knowledge, there has been no systematic comparison of these effects across all three types of dietary FAs (SFAs, MUFAs, and PUFAs) in human monocytes. In this study, we evaluated the effects of 15 of the most common dietary FAs at a range of concentrations. In particular, we measured production of three key pro-inflammatory cytokines (TNF, IL-1β, and IL-6) in human THP-1 monocytes under both normal and inflammation-stimulated conditions. Our results indicate that in the basal condition, SFAs generally act as pro-inflammatory factors, whereas, PUFAs mostly have anti-inflammatory actions (Table 2). The effects of MUFAs vary according to the type of cytokine. Among the six SFAs selected for study, stearic acid was the most potent activator and the only one that could stimulate the release of all three pro-inflammatory cytokines from the resting THP-1 monocytes. Under the LPS treatment condition, all nine selected MUFAs and PUFAs exhibited anti-inflammatory effects within some concentration range. Among these FAs, DHA was the most powerful anti-inflammatory mediator, effectively inhibiting LPS-induced secretion of all three pro-inflammatory cytokines. Furthermore, DPA exerted a global anti-inflammatory effect in both basal and LPS-stimulated conditions. Taken together, these results delineate important composition- and concentration-specific effects of dietary FAs in controlling inflammation.

**Table 2:** Summary of the effects of selected fatty acids on the productions of TNF, IL-1β, and IL-6 in the THP-1 monocytes cultured with and without LPS treatment.

Fatty acid	without LPS			with LPS		
	TNF	IL-1β	IL-6	TNF	IL-1β	IL-6
caprylic acid	**↑**	―	―	N/A	N/A	N/A
capric acid	―	**↑**	―	N/A	N/A	N/A
undecylic acid	―	―	**↑**	N/A	N/A	N/A
lauric acid	―	**↑**	**↑**	N/A	N/A	N/A
palmitic acid	―	**↑**	―	N/A	N/A	N/A
stearic acid	**↑**	**↑**	**↑**	N/A	N/A	N/A
palmitoleic acid	**↑**	**↓**	**↓**	**↓**/**↑**^a^	**↓**/**↑**	**↑**/**↓**
oleic acid	**↑**	―	―	**↓**/**↑**	**↓**/**↑**	**↑**/**↓**
linoleic acid	―	**↓**	**↓**	**↓**/**↓**	**↓**/―	**↑**/**↓**
α-linolenic acid	―	**↓**	**↓**	**↓**/**↓**	**↓**/―	**↑**/**↓**
γ-linolenic acid	**↓**	**↓**	**↓**	**↓**/―	**↓**/**↓**	**↑**/**↓**
arachidonic acid	―	**↓**	**↓**	**↓**/**↓**	**↓**/―	**↑**/**↓**
EPA	―	**↓**	**↓**	**↓**/**↑**	**↓**/**↓**	**↑**/**↓**
DPA	**↓**	**↓**	**↓**	**↓**/**↑**	**↓**/**↓**	**↑**/**↓**
DHA	―	**↓**	**↓**	**↓**/ **↓**	**↓**/**↓**	**↑**/**↓**

**↑**: increase; ↓: decrease; ―: unchanged; ^a^: trend at low concentration/trend at high concentration of FAs.

In our results, stearic acid readily stimulated human THP-1 monocytes to release pro-inflammatory cytokines. Stearic acid is naturally enriched in various dietary sources of fat, such as beef tallow, butterfat, lard, cocoa butter, shea nut oil, and others. The main sources of dietary stearic acid include meat, poultry, fish, eggs, milk products, and oils^51^. Furthermore, pro-inflammatory effects of stearic acid have been demonstrated in the other types of cells. For example, stearic acid upregulates gene expression of TNF, IL-1β, and IL-6 and elicits endoplasmic reticulum stress and apoptosis in triacsin C (long-chain acyl coenzyme A synthetase inhibitor)-treated mouse peritoneal macrophages^52^. In bone marrow-derived macrophages stimulated with macrophage colony-stimulating factor (M-CSF), stearic acid promotes the expression of CD11c, which mediates the production of inflammatory cytokines^53^ and contributes to obesity-associated chronic inflammation and insulin resistance^54–56^. Furthermore, the levels of CD11c induced by stearic acid are higher than those after cells are treated with palmitic acid^56^. Stearic acid also enhances the expression levels of pro-inflammatory markers in microglia, a population of macrophage-like cells in the central nervous system^57^. The stearic acid-induced activation of both macrophages and microglia has been linked to the activation of the toll-like receptor 4/NF-κB signaling pathway^57,58^. Although stearic acid induces pronounced inflammatory responses in macrophages and microglia^56^, it has been reported that hypercholesterolemic postmenopausal women who consume a diet enriched with stearic acid have lower fasting low-density lipoprotein-cholesterol concentrations than those who consume a diet enriched with palmitic acid^59^. It was further postulated that the hypocholesterolemic effect of stearic acid may be due to the reduced overall synthesis of intestinal hydrophobic secondary bile acids. These findings also suggested that dietary SFAs could differentially regulate multiple physiological functions.

DPA and DHA are omega-3 PUFAs that are enriched in fish oil and may potentially be used for treating cardiovascular diseases^60–62^ and metabolic syndrome^63,64^. In our experiments, DPA and DHA exerted anti-inflammatory effects in both resting and LPS-treated THP-1 cells. This result is not surprising because anti-inflammatory effects of omega-3 PUFAs are widely recognized. Reported actions of omega-3 PUFAs include inhibitory effects on T cell reactivity, leukocyte chemotaxis, and inflammatory cytokine production^65^. These effects are at least partly regulated by different omega-3 PUFAs-derived specific pro-resolving mediators, which are bioactive metabolites produced by fatty acid oxygenases^66^. In the group of four tested omega-3 PUFAs (α-linolenic acid, EPA, DPA, DHA), we noticed a positive relationship between the number of unsaturated bonds and the anti-inflammatory effect size. DHA (C22:6) exhibited the strongest effect, followed by DPA (C22:5) and EPA (C20:5), and then α-linolenic acid (C18:3). Stronger anti-inflammatory effects of DHA compared to other omega-3 PUFAs has also been reported in previous studies using THP-1-derived macrophages^67^. Furthermore, a recently published randomized controlled trial study revealed that DHA has a broader suppressive effect on pro-inflammatory cytokines than EPA in humans with chronic inflammation^68^. Interestingly, we found that almost all of the MUFAs and PUFAs we tested increased the LPS-induced production of TNF and IL-1β when treated at 500 µM, while the 10 µM dose increased production of IL-6 in the THP-1 monocytes. These results suggest that the inhibitory effects of MUFAs and PUFAs on LPS-induced cytokine secretion follow either U-shaped (TNF and IL-1β) or inverted U-shaped (IL-6) dose–response curves. However, these types of dose–response curves were not observed in THP-1 cells in a resting state. Although the underlying mechanisms remain unclear, these findings suggest that there is a particular dosage window in which MUFAs/PUFAs act on monocytes in an anti-inflammatory manner. Similar non-linear dose responses have also been observed for vitamin and mineral supplements on health-related outcomes. While it is widely known that vitamins and minerals are essential for maintaining normal physiological functions, excessive supplementation can lead to toxicity^69^. Omega-3 FAs are also frequently consumed as dietary supplements due to their beneficial effects in a broad range of health conditions^70^. As such, it will be crucial to determine the correlation between circulating levels of dietary omega-3 FAs and pro-inflammatory cytokines in humans in order to recommend optimal doses for supplements to reduce systemic inflammation. These findings also highlight the need for individuals to be mindful of systemic inflammation effects when consuming high doses of omega-3 FAs for other purposes.

In conclusion, we found that SFAs generally stimulated secretion of pro-inflammatory cytokines in resting THP-1 cells, with stearic acid being the most potent species. Meanwhile, MUFAs and PUFAs inhibited LPS-induced secretion of pro-inflammatory cytokines. These inhibitory effects followed either U-shaped (TNF and IL-1β) or inverted U-shaped (IL-6) dose–response curves, implying that distinct dose windows exist for anti-inflammatory effects of dietary MUFAs/PUFAs. Among the MUFAs and PUFAs that we tested, DHA exhibited the largest number of double bonds and was found to be the most potent anti-inflammatory compound. Together, our findings reveal that dietary FA chemical compositions (double bond number) and concentrations (nonlinear effects) are key factors in the intricate regulation of monocyte-mediated inflammation.

## Materials and methods

### THP-1 cell cultures

THP-1 cells were obtained from the Bioresource Collection and Research Center of Taiwan (Cat. #: 60430, Hsinchu, Taiwan) and cultured in ATCC-modified RPMI 1640 medium (Cat. #: A1049101, Thermo Fisher Scientific, Waltham, MA, USA) supplemented with 10% fetal bovine serum with endotoxin less than 0.05 EU/mL (Lot# VP2002200, Cat. #: TMS-013-BKR, Merck-Millipore, Burlington, MA, USA), 0.05 mM β-mercaptoethanol (Cat. #: 31350010, Thermo Fisher Scientific), and penicillin–streptomycin (Cat. #: 15140122, Thermo Fisher Scientific). Cultures were kept in a humidified atmosphere of 5% CO_2_ and 95% air at 37 °C. Subcultures were carried out when cell concentration reached 8 × 10^5^ cells/mL by adding fresh medium. Cell density was kept under 1 × 10^6^ cells/mL during this study.

### Preparation of FAs

The 15 selected FAs are listed in Table 1. Caprylic acid (Cat. #: C2875, Sigma-Aldrich, St. Louis, MO, USA), capric acid (Cat. #: C1875, Sigma-Aldrich), undecylic acid (Cat. #: U0004, Tokyo Chemical Industry, Chuo-ku, Tokyo, Japan), lauric acid (Cat. #: W261408, Sigma-Aldrich), myristic acid (Cat. #: M3128, Sigma-Aldrich), palmitoleic acid (Cat. #: P9417, Sigma-Aldrich), stearic acid (Cat. #: S4751, Sigma-Aldrich), oleic acid (Cat. #: O1008, Sigma-Aldrich), linoleic acid (Cat. #: L1376, Sigma-Aldrich), α-linolenic acid (Cat. #: L2376, Sigma-Aldrich), γ-linolenic acid (Cat. #: L2378, Sigma-Aldrich), arachidonic acid (Cat. #: A0781, Tokyo Chemical Industry), eicosapentaenoic acid (EPA, Cat. #: E0441, Tokyo Chemical Industry), docosapentaenoic acid (DPA, Cat. #: D1797, Sigma-Aldrich), and docosahexaenoic acid (DHA, Cat. #: D2534, Sigma-Aldrich) were dissolved in ethanol (Cat. #: 1.00983, Sigma-Aldrich) to make a working solution of 200 mM. The working solution was diluted ten times in PBS (Cat. #: 10010023, Thermo Fisher Scientific) containing 20% FA-free bovine serum albumin (BSA, Cat. #: A8806, Sigma-Aldrich) and incubated at 55 °C for one hour to make a 20 mM BSA-conjugated FA solution. However, when the palmitic acid–ethanol solution was mixed with bovine serum albumin, a noticeable precipitate formed. To resolve this issue, palmitic acid (Cat. #: P0500, Sigma-Aldrich) was instead dissolved in 0.01 N NaOH (Cat. #: S8045, Sigma-Aldrich; 20 mM), combined with PBS (Cat. #: 10010023, Thermo Fisher Scientific) containing 5% FA-free bovine serum albumin (Cat. #: A8806, Sigma-Aldrich) in a 2:3 ratio, and incubated at 55 °C for an hour to produce an eight mM BSA-conjugated palmitic acid stock solution. The vehicle solution for each FA was prepared using the same process.

### Cell treatments

THP-1 cells were centrifuged at 200 × g for 5 min and the pellet was suspended in FBS-free culture media to make a concentration of 9.5 × 10^5^ cells/mL before being transferred into 6-well plates (one mL/well). After a 16-h incubation period, the THP-1 cultures were treated with different doses of FAs (final concentrations: 0, 10, 50, 100, 200, and 500 µM) either alone or in combination with 100 ng/mL of LPS (from Escherichia coli O55:B5, Cat. #: L2880, Sigma-Aldrich) for 24 h. Vehicle controls contained the same amounts of BSA and ethanol/NaOH used in different doses of FA experiments. At the end of the incubation, cells were collected by gently flushing. Twenty µL of the cell mixture was used to assess the cell viability through trypan blue dye exclusion staining and counting using a hemocytometer as previously described^71,72^. The rest of the mixtures were centrifuged at 200 × g for five min and the conditioned media were harvested for the determination of cytokine concentrations. Sample size = nine in each experiment.

### Measurements of pro-inflammatory cytokines

The concentrations of TNF, IL-1β, and IL-6 in the conditioned media were determined by commercial human TNF (Cat. #: 550610, Becton, Dickinson and Company, Franklin Lakes, NJ, USA), IL-1β (Cat. #: 557966, Becton, Dickinson and Company), and IL-6 (Cat. #: 550799, Becton, Dickinson and Company) ELISA kits following the manufacturer’s instructions.

### Statistical analysis

All numerical data are expressed as mean ± standard deviation. Statistical analyses and graph plotting were performed using the Prism software (v. 7.0a, GraphPad Software Inc., San Diego, CA, USA). Significance was set at *p* < 0.05. One-way ANOVA followed by Dunnett’s multiple comparisons was used to analyze differences between independent groups.

## Supplementary Information

Supplementary Table 1.

## Data Availability

All datasets generated or analyzed in this study were included in the published article. Detailed datasets supporting the current study are available from the Corresponding Authors upon request.
