# Supplementary material for: Dietary fatty acids differentially affect secretion of pro-inflammatory cytokines in human THP-1 monocytes
Source: Sci Rep. 2023 Apr 4;13:5511. doi: 10.1038/s41598-023-32710-5 (PMC10073224; doi:10.1038/s41598-023-32710-5)
Supplement: Supplementary file 1 — Supplementary Table 1. [file 41598_2023_32710_MOESM1_ESM.docx]

**Supplementary Table 1.** Results of THP-1 cell viability (%, compared to respective 0 μM group) in each experiment.

| ***THP-1 cultures without LPS stimulation*** | | | | | | |
| --- | --- | --- | --- | --- | --- | --- |
|  | **0 μM** | **10μM** | **50μM** | **100μM** | **200μM** | **500μM** |
| caprylic acid | 100.00 ± 13.61 | 97.59 ± 13.99 | 89.42 ± 10.89 | 95.24 ± 15.18 | 89.36 ± 12.80 | 85.84 ± 12.77 |
| capric acid | 100.00 ± 14.12 | 88.71 ± 12.36 | 88.23 ± 7.23 | 93.20 ± 4.52 | 91.44 ± 11.36 | 88.90 ± 14.06 |
| undecylic acid | 100.00 ± 10.39 | 91.23 ± 10.00 | 91.74 ± 14.82 | 87.19 ± 10.80 | 96.47 ± 8.22 | 92.62 ± 9.10 |
| lauric acid | 100.00 ± 10.20 | 90.10 ± 11.26 | 94.77 ± 14.50 | 89.85 ± 10.90 | 90.28 ± 10.05 | 84.75 ± 8.99 |
| palmitic acid | 100.00 ± 11.44 | 88.66 ± 15.78 | 96.07 ± 17.50 | 85.98 ± 15.74 | 77.52 ± 17.54 | 82.77 ± 20.33 |
| stearic acid | 100.00 ± 19.12 | 87.63 ± 15.94 | 78.80 ± 21.27 | 82.54 ± 16.56 | 72.36 ± 17.76 | 82.86 ± 19.14 |
| palmitoleic acid | 100.00 ± 10.23 | 84.46 ± 13.61 | 83.18 ± 18.45 | 84.89 ± 14.51 | 80.18 ± 14.48 | 77.31 ± 19.52 |
| oleic acid | 100.00 ± 10.34 | 91.35 ± 12.60 | 83.77 ± 12.58 | 84.16 ± 13.43 | 87.09 ± 19.27 | 79.84 ± 15.41 |
| linoleic acid | 100.00 ± 13.05 | 102.80 ± 10.56 | 111.00 ± 14.22 | 94.51 ± 13.62 | 103.70 ± 16.09 | 98.37 ± 9.55 |
| α-linolenic acid | 100.00 ± 8.04 | 93.79 ± 15.79 | 94.57 ± 10.14 | 92.66 ± 15.05 | 92.66 ± 15.05 | 83.49 ± 23.05 |
| γ-linolenic acid | 100.00 ± 12.66 | 97.35 ± 22.75 | 94.99 ± 8.17 | 85.74 ± 17.50 | 81.95 ± 17.38 | 84.60 ± 21.08 |
| arachidonic acid | 100.00 ± 18.27 | 97.18 ± 11.10 | 93.13 ± 20.64 | 92.99 ± 15.82 | 79.32 ± 26.87 | 92.48 ± 22.05 |
| EPA | 100.00 ± 12.57 | 96.53 ± 21.66 | 98.61 ± 23.23 | 108.20 ± 12.03 | 92.99 ± 29.78 | 96.92 ± 23.29 |
| DPA | 100.00 ± 12.70 | 92.12 ± 13.63 | 89.02 ± 9.73 | 82.01 ± 12.41 | 84.03 ± 10.75 | 81.50 ± 25.41 |
| DHA | 100.00 ± 13.12 | 98.08 ± 12.20 | 90.91 ± 10.83 | 91.65 ± 25.30 | 97.04 ± 7.40 | 84.55 ± 21.46 |
| ***THP-1 cultures with LPS stimulation*** | | | | | | |
|  | **0 μM** | **10μM** | **50μM** | **100μM** | **200μM** | **500μM** |
| palmitoleic acid | 100.00 ± 23.38 | 102.60 ± 19.96 | 95.57 ± 26.53 | 105.20 ± 11.44 | 92.32 ± 22.20 | 78.92 ± 21.36 |
| oleic acid | 100.00 ± 17.94 | 108.50 ± 12.68 | 79.63 ± 19.51 | 93.25 ± 25.57 | 93.87 ± 27.91 | 92.52 ± 18.52 |
| linoleic acid | 100.00 ± 19.62 | 83.94 ± 18.93 | 96.08 ± 22.17 | 94.31 ± 17.81 | 98.99 ± 17.83 | 84.96 ± 23.04 |
| α-linolenic acid | 100.00 ± 9.17 | 85.93 ± 6.57 | 84.56 ± 16.07 | 79.34 ± 16.14 | 79.23 ± 17.67 | 80.82 ± 21.92 |
| γ-linolenic acid | 100.00 ± 18.45 | 120.80 ± 18.10 | 94.67 ± 15.08 | 82.96 ± 19.63 | 82.29 ± 30.78 | 77.10 ± 23.98 |
| arachidonic acid | 100.00 ± 16.35 | 93.81 ± 9.52 | 91.02 ± 19.90 | 90.14 ± 10.13 | 98.36 ± 14.66 | 82.68 ± 20.71 |
| EPA | 100.00 ± 16.32 | 103.40 ± 9.52 | 109.40 ± 9.74 | 96.99 ± 18.00 | 93.84 ± 12.53 | 84.14 ± 24.42 |
| DPA | 100.00 ± 22.04 | 109.40 ± 26.04 | 100.90 ± 30.91 | 99.29 ± 20.44 | 84.74 ± 25.46 | 95.44 ± 23.84 |
| DHA | 100.00 ± 7.54 | 86.68 ± 17.22 | 94.20 ± 25.68 | 86.93 ± 19.58 | 101.40 ± 12.85 | 82.12 ± 26.56 |

Data are presented as mean ± standard deviation and analyzed with Dunnett’s multiple comparisons after one-way ANOVAs. n = 9.
